# Synaptic Impairment in Layer 1 of the Prefrontal Cortex Induced by Repeated Stress During Adolescence is Reversed in Adulthood

**DOI:** 10.3389/fncel.2015.00442

**Published:** 2015-11-12

**Authors:** Ignacio Negrón-Oyarzo, Alexies Dagnino-Subiabre, Pablo Muñoz Carvajal

**Affiliations:** ^1^Centro Interdisciplinario de Neurociencia de Valparaíso, Universidad de ValparaísoValparaíso, Chile; ^2^Laboratorio de Neurobiología y Conducta, Centro de Neurobiología y Plasticidad Cerebral, Instituto de Fisiología, Facultad de Ciencias, Universidad de ValparaísoValparaíso, Chile; ^3^Departamento de Psiquiatría, Facultad de Medicina, Centro Interdisciplinario de Neurociencia, Pontificia Universidad Católica de ChileSantiago, Chile; ^4^Centro Interdisciplinario de Innovación en Salud, Escuela de Medicina, Facultad de Medicina, Universidad de ValparaísoValparaíso, Chile

**Keywords:** repeated stress, synaptic plasticity, prefrontal cortex, adolescence, long-term depression

## Abstract

Chronic stress is a risk factor for the development of psychiatric disorders, some of which involve dysfunction of the prefrontal cortex (PFC). There is a higher prevalence of these chronic stress-related psychiatric disorders during adolescence, when the PFC has not yet fully matured. In the present work we studied the effect of repeated stress during adolescence on synaptic function in the PFC in adolescence and adulthood. To this end, adolescent Sprague-Dawley rats were subjected to seven consecutive days of restraint stress. Afterward, both synaptic transmission and short- and long-term synaptic plasticity were evaluated in layer 1 of medial-PFC (mPFC) slices from adolescent and adult rats. We found that repeated stress significantly reduced the amplitude of evoked field excitatory post-synaptic potential (fEPSP) in the mPFC. Isolation of excitatory transmission reveled that lower-amplitude fEPSPs were associated with a reduction in α-amino-3-hydroxy-5-methyl-4-isoxazolepropionic acid (AMPA) receptor-mediated transmission. We also found that repeated stress significantly decreased long-term depression (LTD). Interestingly, AMPA/kainate receptor-mediated transmission and LTD were recovered in adult animals that experienced a three-week stress-free recovery period. The data indicates that the changes in synaptic transmission and plasticity in the mPFC induced by repeated stress during adolescence are reversed in adulthood after a stress-free period.

## Introduction

Stress is a biological response that allows adaptation to environmental threats (Rodrigues et al., 2009). However, when threats are excessively intense and persistent, the stress response can be maladaptive (McEwen, 2002). Under these conditions, chronic stress becomes a well-established risk factor for the development of mood disorders (Taffet and Bernardini, 2003; Hammen, 2005; Shin and Liberzon, 2010). However, the underlying mechanism by which chronic stress is associated with the development of these disorders is not completely understood. The prefrontal cortex (PFC), a brain region involved in controlling high-level executive functions (Fuster, 2001; Miller and Cohen, 2001), displays functional impairment in patients suffering mood disorders (Drevets et al., 1997; Johnstone et al., 2007). This suggests that chronic stress induces alterations in the PFC related to behavioral dysfunction (Liston et al., 2006; Miracle et al., 2006; Dias-Ferreira et al., 2009; Holmes and Wellman, 2009).

The pathophysiology of these stress-related alterations may be related to synaptic impairment (Duman, 2002; Goto et al., 2010; Christoffel et al., 2011). For example, in the medial-PFC (mPFC), the rodent equivalent to the primate PFC (Uylings et al., 2003), the most documented effects of chronic stress are dendritic atrophy and reduction of dendritic spines in the apical tufts of pyramidal neurons (Cook and Wellman, 2004; Radley et al., 2004, 2005, 2008; Brown et al., 2005; Liu and Aghajanian, 2008). These alterations are paralleled with impairment of mPFC-related behaviors (Liston et al., 2006; Dias-Ferreira et al., 2009). At the physiological level, glutamatergic synaptic transmission and plasticity in the mPFC, which are crucial for mPFC-related cognitive and affective processes (Goto et al., 2010; Popoli et al., 2011; Graybeal et al., 2012), are also affected by chronic stress (Lisman et al., 1998; Burgos-Robles et al., 2007). For example, *in vivo* and *in vitro* activity-dependent synaptic plasticity is affected by chronic stress (Abramets et al., 2004; Cerqueira et al., 2007; Goldwater et al., 2009; Judo et al., 2010; Quan et al., 2011). Interestingly, to our knowledge no studies have examined the effect of chronic stress on superficial layers of the mPFC where apical dendritic atrophy of pyramidal neurons occurs (Radley et al., 2004, 2005, 2008; Liu and Aghajanian, 2008).

Adolescence is characterized by an increased prevalence of chronic stress-related disorders (Romeo and McEwen, 2006; Paus et al., 2008). Adolescent animals display a higher stress response than adults (Pruessner et al., 2005; McCormick et al., 2010), which suggests adolescents are more sensitive to chronic stress (Romeo and McEwen, 2006). During adolescence, the PFC undergoes profound neuronal modifications that contribute to full maturity in adulthood, which is paralleled by the complete development of higher cognitive functions (Kolb et al., 2010). Considering that neuronal maturation in the PFC occurs largely during adolescence (Markham et al., 2007, 2013; Kolb et al., 2010), it is likely that the effects of chronic stress on the PFC are more pronounced during this stage, but reversible in adulthood after a stress-free period. However, it is unknown whether behavioral and neurophysiological alterations induced by chronic stress in the PFC are reversed in adulthood.

We recently showed that rats stressed during adolescence display impairment in the recall of extinction of conditioned fear, a mPFC-related behavioral task (Quirk et al., 2000; Quirk and Mueller, 2008) that is recovered at adulthood (Negrón-Oyarzo et al., 2014). This recovered behavior is paralleled by the recovery of basal synaptic transmission in the mPFC (Negrón-Oyarzo et al., 2014). In the present work we complemented our previous study by evaluating the effect of chronic stress during adolescence in excitatory and inhibitory transmission, short-term synaptic plasticity, and activity-dependent synaptic plasticity in layer 1 of the mPFC.

## Materials and Methods

### Animals

Male Sprague-Dawley rats were housed in groups of 3–4 per home cage under a 12 h light / dark cycle (lights at 8:00 am), *ad libitum* access to food (LabDiet) and water, and at a room temperature of (21 ± 1°C). All animals were handled and weighed daily after weaning. All procedures related to animal experimentation were in accordance with NIH guidelines and were approved by the Institutional Animal Ethics Committee of the Universidad de Valparaíso, Chile. Efforts were made to minimize the number of animals used and their suffering.

### Experimental Design and Restraint Stress Protocol

Adolescence in male rats is considered to last from post-natal day (PND) 35–55 (Ojeda and Skinner, 2006). Once the rats reached PND42, they were randomly assigned to control (*n* = 8) or stress (*n* = 8) group. Control animals were housed in a separate room and not subjected to any type of stress. Stress group animals were subjected to restraint stress in their home cages for 3 h per day for seven consecutive days (from PND 42–49) in a cylindrical acrylic restrainer (8 cm in diameter × 22 cm long). To evaluate synaptic function at adolescence and adulthood, four animals per group were subjected to electrophysiological experiments 1 day (PND50, adolescents) or 21 days (PND70, adults) after the end of the stress protocol. The percentage of body weight gain (net change in weight in grams × 100/weight at the beginning of the stress protocol *n* = 8 per group) and adrenal weight (wet weight of adrenal glands in mg × 100/body weight in grams *n* = 6 per group) were measured during the stress protocol to monitor physiological stress response (Ulrich-Lai et al., 2006).

### Electrophysiology

Control and stressed adolescent and adult rats were anesthetized with halothane and sacrificed by decapitation. Brains were quickly removed and submerged in cold dissection buffer containing 300 mM sucrose; 6 mM MgSO_4_; 4 mM KCl, 1 mM Na_2_HPO_4_, 0.5 mM CaCl_2_, 26 mM NaHCO_3_, and 10 mM D-glucose, and constantly bubbled with 95%O_2_/5%CO_2_. Coronal 400 μm slices containing the PL (2.20–3.70 mm from Bregma; Paxinos and Watson, 1998) were sectioned using a vibratome (Pelco) in constantly bubbled cold dissection buffer. The slices were then transferred to a holding chamber immersed in artificial cerebrospinal fluid (ACSF) containing 124 mM NaCl; 4 mM KCl; 1 mM Na_2_HPO_4_; 26 mM NaHCO_3_; 1 mM MgCl_2_; 2 mM CaCl_2_ and 10 mM D-glucose constantly bubbled with 95%O_2_/5%CO_2_, and maintained undisturbed at 28 ± 0.5°C for at least 2 h. All electrophysiological experiments were conducted blind.

### Recording

Slices containing mPFC were transferred individually to an immersion-type recording chamber, which was continuously perfused with ACSF at 2 ml/min and 28 ± 1°C. A bipolar concentric tungsten-stimulating electrode (FHC) was positioned in layer 1 of the prelimbic cortex, the dorsal part of the mPFC (Uylings et al., 2003). Field excitatory post-synaptic potentials (fEPSP) were evoked by applying constant-current monophasic square pulses (200 μs) at an intensity range of 10–50 μA and a frequency of 0.033 Hz with a stimulus isolator (model SIU91A, Cygnus Technology, Delaware Water Gap, PA, USA). The evoked fEPSPs were recorded with a borosilicate pipette filled with 2 M NaCl placed in layer 1 at approximately 100 μm from the stimulating electrode. Recordings were amplified 1000×, bypass filtered at 1 Hz–5 kHz (amplifier model 1700, A-M system amplifier; Sequim, WA, USA), and acquired on a PC computer using an analog/digital converter interface (Model BNC-2090, National Instruments). Recordings were acquired and analyzed off-line using IgorPro software.

Input-output curves were generated for the slices to evaluate basal synaptic transmission. We considered the average of three pulses delivered at 0.33 Hz at each intensity in the range of 0–50 μA in steps of 10 μA. Synaptic strength was determined by measuring the peak amplitude of the negative component of the fEPSPs include reference by Cauller and Connors (1994), which is already included in the reference section. To evaluate short-term synaptic plasticity, we applied paired-pulse stimulation (PPS) at inter-stimulus intervals (ISI) of 20, 40, 80, and 120 ms. The paired-pulse ratio (PPR) was calculated as the amplitude of the fEPSP elicited by the second pulse divided by the amplitude elicited by the first (PPR = A2/A1). Two simulation protocols were applied to induce long-term cortical potentiation (Aroniadou and Keller, 1995; Kirkwood et al., 1997): high-frequency stimulation (HFS; single train of 100 pulses delivered at 50 Hz) and theta-burst stimulation (TBS; four trains delivered at 0.05 Hz, each train composed of 10 bursts delivered at 10 Hz, and each burst composed of four pulses delivered at 100 Hz). Long-term depression (LTD) was induced by a single train of 900 pulses delivered at 1 Hz. LTP and LTD were expressed as the percentage of the fEPSP amplitude after the application of the stimulation protocol with respect to baseline.

### Pharmacology

To isolate the excitatory component of the fEPSPs, the slices were incubated in normal ACSF containing 1 μM picrotoxin (PTX), a gamma-aminobutyric acid (GABA)-type A receptor (GABA_A_R) antagonist. To isolate the AMPA/kainate receptor-mediated fEPSP, the slices were incubated in normal ACSF containing 50 μMD-2-amino-5-phosphonovaleric acid (APV), a N-Methyl-D-aspartate (NMDA) receptor (NMDAR) antagonist, and 1 μM PTX. To isolate the NMDAR-mediated fEPSP, the slices were incubated in ACSF without Mg^2+^ containing 5 μM 6-cyano-7-nitro-quinoxaline-2,3-dione (CNQX), an AMPA/kainate antagonist, and 1 μM PTX.

### Statistic Analysis

Data from body weight gain, fEPSP amplitude in the input-output curves, PPR and fEPSP amplitudes as percentages of the baseline in the LTP and LTD were analyzed with a two-way repeated-measures ANOVA, followed by a Bonferroni *post hoc* test. Adrenal weight data were analyzed with the Mann-Whitney U-test. A p-value of 0.05 or less was considered statistically significant. Data are presented as means ± S.E.M.

## Results

### Stress Markers

**Figure 1:**
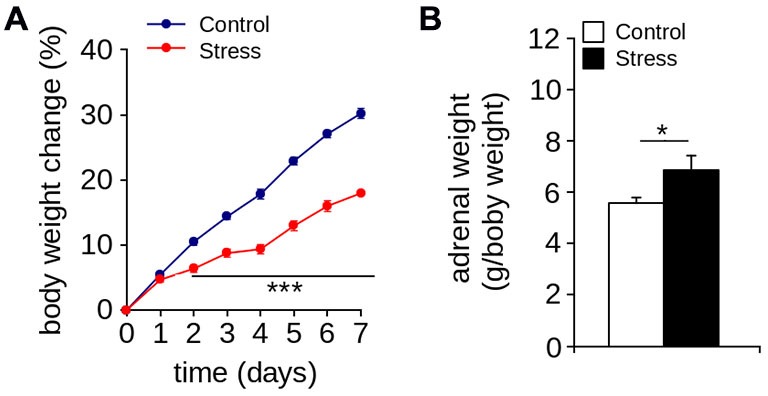
**Stress markers. (A)** Body weight gain (***p < 0.001; Bonferroni *post hoc* test; *n* = 8 per group). **(B)** Adrenal weight of control and stressed animals was measured one day after the stress protocol (*p < 0.05; Mann-Whitney U-test; *n* = 6 per group). Values are mean ± SEM.

**Figure 2:**
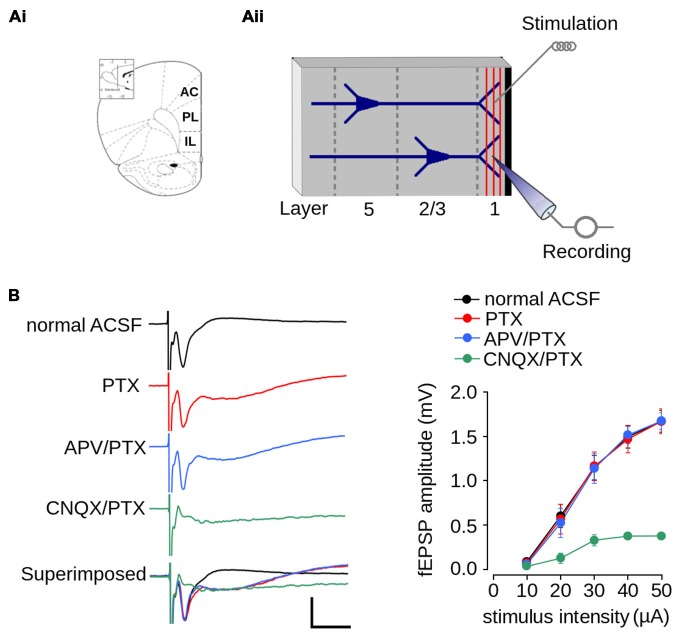
**Excitatory field post-synaptic potential recorded in layer 1 of mPFC. (Ai)** Schematic diagram of the rat mPFC (from Paxinos and Watson, 1998; AC, cingulate cortex; PL, prelimbic cortex; IL, infralimbic cortex); **(Aii)** Schematic diagram of the location of the recording and stimulating electrodes in layer 1 of mPFC. **(B)** Pharmacologically isolated components of the fEPSPs recorded in layer 1. Left panel, representative traces of the isolated fEPSPs evoked at the same stimulus intensity. From top to bottom: response in normal ACSF; in ACSF containing 1 μM picrotoxin (PTX); in ACSF containing 1 μM PTX and 50 μM APV; in ACSF devoid Mg^2+^ containing 5 μM CNQX and 1 μM PTX; and superimposed traces (scale bar: 1 mV; 10 ms). Right panel: summarized input-output curves plotting the average fEPSP amplitude at different stimulus intensities in standard ACSF or in the presence of the indicated drugs in the perfusion bath. Data are expressed as mean ± SEM (*n* = 4 animals, 17-23 slices).

Figure 1A show that seven days of restraint decreased the percentage of weight gain. The body weight of control and stressed rats increased over time (effect of days: *F*_(7,122)_ = 365.4; *p* < 0.001; *n* = 8 per group). However, repeated application of the restraint protocol significantly reduced body weight gain (effect of group: *F*_(1,220)_ = 401.6; *p* < 0.0001; interaction between the passage of days and stress protocol: *F*_(7,122)_ = 15.05; *p* < 0.0001). We also found that seven days of restraint significantly increased adrenal weight (Figure 2B; *Control* = 6.47 ± 0.34 g/body weight in g; *Stress* = 7.97 ± 0.54 g/body weight in g; *p* < 0.05; *n* = 6 per group). These results indicate that seven days of restraint was effective in activating a chronic stress response.

### Characterization of Synaptic Transmission and Plasticity in Layer 1 of the mPFC

We first characterized synaptic transmission in layer 1 of the mPFC of non-stressed animals. Figure 2Ai shows an schematic diagram of the rat mPFC. Figure 2Aii shows a schematic diagram with the arrangement of the recording and stimulating electrodes in layer 1 of the mPFC. Electrical stimulation of layer 1 evoked a negative extracellular wave in the same layer, which likely corresponds to the fEPSP. The recorded fEPSP had maximum amplitude of 1.67 ± 0.12 mV and a latency-peak of 4.031 ± 0.57 ms at 50 μA of stimulus intensity (Figure 2B). The short latency of this response, together with the absence of long-latency responses, strongly suggests that fibers stimulated in layer 1 activated superficial dendrites monosynaptically.

Given that superficial layers of mPFC contain GABAergic inhibitory interneurons (Gabbott et al., 1997), we evaluated the contribution of inhibitory transmission on the amplitude of evoked fEPSPs. The application of PTX did not affect fEPSP amplitudes (Figure 2B; effect of drug: *F*_(1,90)_ = 0.022; *p* = 0.881), suggesting that there were no significant inhibitory transmissions in the evoked fEPSPs. However, the application of PTX resulted in a negative field potential following the fEPSP (Figure 2B), which suggests that the recovery phase of the fEPSPs is influenced by GABAergic-inhibitory transmission.

Excitatory synaptic transmission in layer 1 of mPFC is mainly glutamatergic (Hirsch and Crepel, 1990; Bai et al., 2014). Accordingly, we examined the contribution of both AMPA/kainate and NMDA glutamatergic receptors to the amplitude of the evoked fEPSP. We first isolated the AMPA/kainate receptor-mediated fEPSP by superfusing ACSF containing APV in the presence of PTX. As shown in Figure 2B, the application of APV and PTX did not affect fEPSPs amplitude (effect of drug: *F*_(1,90)_ = 0.061; *p* = 0.804). We then isolated the NMDAR-mediated fEPSPs by superfusing AMPA/kainate inhibitor CNQX and PTX in ACSF lacking Mg^2+^. Treatment with both agents resulted in the almost complete abolition of post-synaptic response. However, strong stimulation (50 μA) evoked a field potential of lower amplitude than that evoked in ACSF (ACSF = 1.67 ± 0.12 mV; ACSF + CNQX = 0.378 ± 0.04 mV; Figure 2B), which is likely a NMDAR-mediated response.

**Figure 3:**
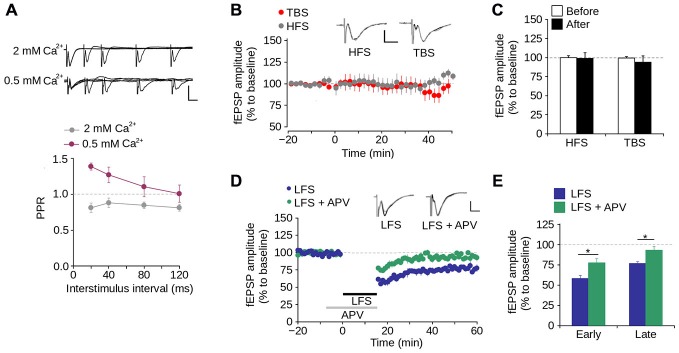
**Synaptic plasticity in layer 1 of the mPFC. (A)** Upper panel, Superimposed representative fEPSP traces evoked by paired-pulse stimulation (PPS) at different inter-stimulus intervals (ISI) between 20–120 ms in ACSF containing either 2 mM or 0.5 mM [Ca^2+^] (Scale bar: 1 mV; 10 ms). Lower panel, quantification of paired-pulse ratio (PPR) at different ISI for 2 and 0.5 mM [Ca^2+^] in the ACSF. **(B)** Time lapse of the average fEPSP amplitude expressed in percentage of the baseline before theta-burst stimulation (TBS) or high-frequency stimulation (HFS). Inset: superimposed representative fEPSP traces evoked before and after TBS or HFS (Scale bar: 0.5 mV; 5 ms). **(C)** Bar chart of the average change of fEPSP amplitude expressed as percentage ten minutes before and 40 min after the application of either TBS or HFS. **(D)** Time lapse of normalized fEPSP amplitude expressed as percentage of the baseline. The application of low-frequency stimulation protocol (LFS; 900 pulses at 1 Hz, indicated by the black bar) depressed the fEPSP amplitude. This depression was blocked by the application of 50 μM APV in the perfusion bath, ten minutes before and during the application of the LFS protocol (indicated by the gray bar). Inset: superimposed representative traces recorded before and after LFS for both normal and APV conditions (Scale bar: 0.5 mV; 5 ms). **(E)** Bar chart of the average normalized fEPSP amplitude recorded during the first 10 min (early-LTD) and last 10 min (late-LTD) after the application of the LFS protocol (*p < 0.05; Bonferroni *post hoc* comparison after ANOVA; *n* = 9 per group). Data are expressed as mean ± SEM (*n* = 4 animals, 17-23 slices).

We then evaluated short-term synaptic plasticity using the PPS protocol. As shown in Figure 3A, at ISIs between 20–120 ms at a normal Ca^2+^ concentration in the perfusion medium (2 mM), the fEPSP evoked by the second pulse had a lower amplitude than that evoked by the first pulse, with PPR values <1.0. This indicates that synapses of layer 1 have paired-pulse depression. To evaluate the contribution of Ca^2+^ to short-term synaptic plasticity, we tested PPS at a low Ca^2+^ concentration (0.5 mM) and observed that the amplitude of the fEPSP evoked by the second pulse was higher than that evoked by the first pulse. This resulted in PPR values >1.0, indicative of paired-pulse facilitation (Figure 3A). These results indicate that short-term synaptic plasticity in layer 1 of the mPFC is dependent on the Ca^2+^ concentration, suggesting a mechanism mediated by presynaptic neurotransmitter release.

To evaluate long-term synaptic plasticity, we examined whether the synapses in layer 1 of the mPFC display either LTP or LTD. We found that neither HFS nor TBS induces LTP in layer 1 (Figures 3B,C). In contrast, LFS significantly reduced the fEPSP amplitude 40–60 min after its application (Figures 3D,E; 23.41 ± 2.42% of the baseline; *p* < 0.001). Superfusing APV into the bath produced a significant difference in the fEPSP amplitude compared to LFS condition, in both early-phase (first 10 min after LFS application; LFS = 58.02 ± 3.91%; LFS + APV = 77.57 ± 5.23%; *p* < 0.001) and late-phase LTD (last 10 min after LFS application; LFS = 76.59 ± 2.42%; LFS + APV = 93.90 ± 4.45%; *p* < 0.001; Figures 3D,E). These data indicate that under our working conditions NMDAR-dependent LTD (but not LTP) can be induced in layer 1 of the mPFC.

### Repeated Stress Reduced Excitatory Synaptic Transmission in the mPFC During Adolescence that was Reversed in Adulthood

**Figure 4:**
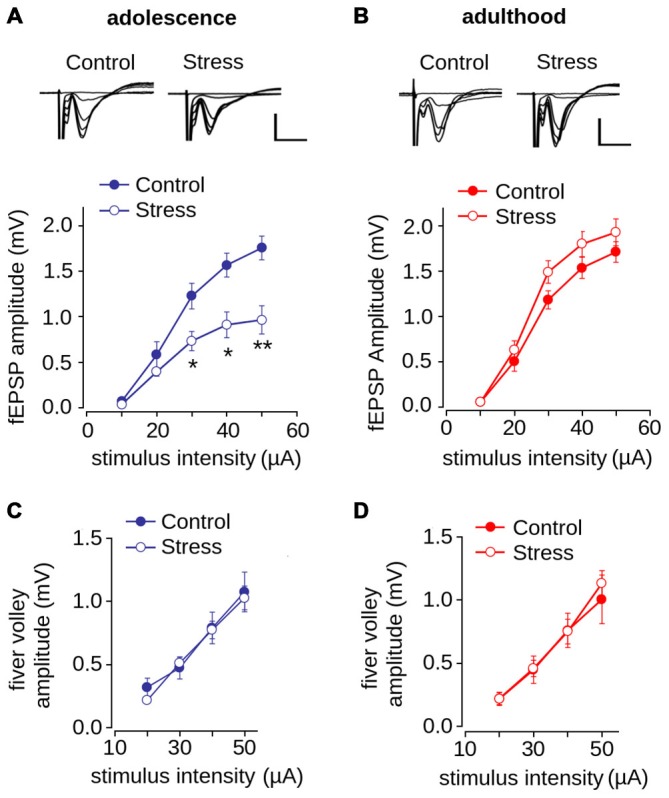
**Effect of repeated restraint stress during adolescence on synaptic transmission in layer 1 of mPFC is reversed in adulthood. (A,B)** Input-output relationship of the average fEPSP amplitude as a function of stimulation intensity measured in adolescence **(A)** and adulthood **(B)**. Significant difference between control and stress groups (*p < 0.05; **p < 0.01; Bonferroni *post hoc* test). Inset: representative superimposed traces of the fEPSP evoked at different stimulus intensities for control and stress groups. Scale bar: 1.0 mV; 5 ms. **(C,D)** Input-output relationship of the average fiber volley amplitude as a function of stimulation intensity measured during adolescence **(C)** and adulthood **(D)**. Data are expressed as mean ± SEM (*n* = 4 animals, 17-23 slices).

We next evaluated the effect of repeated stress during adolescence on synaptic transmission in the mPFC in adolescence and adulthood. We measured fEPSP amplitude evoked by different intensities of electrical stimulation and found as shown previously (Negrón-Oyarzo et al., 2014), that the amplitude of stressed adolescent animals was significantly lower than that of control animals (Figure 4A; effect of repeated stress: *F*_(1,138)_ = 21.39, *p* < 0.001). Bonferroni *post hoc* comparison revealed that the fEPSP amplitudes of stressed animals were significantly lower than those of the controls at stimulus intensities of 30, 40 (*p* < 0.01) and 50 μA (*p* < 0.001). In contrast, we did not find significant differences between adult control and stressed rats (Figure 4B; *F*_(1,256)_ = 1.43; *p* = 0.231). We found no differences between groups in the amplitude of the fiber volley in either adolescence (*F*_(1,102)_ = 0.156; *p* = 0.693) or adulthood (*F*_(1,136)_ = 1.184; *p* = 0.278; Figures 4C,D).

**Figure 5:**
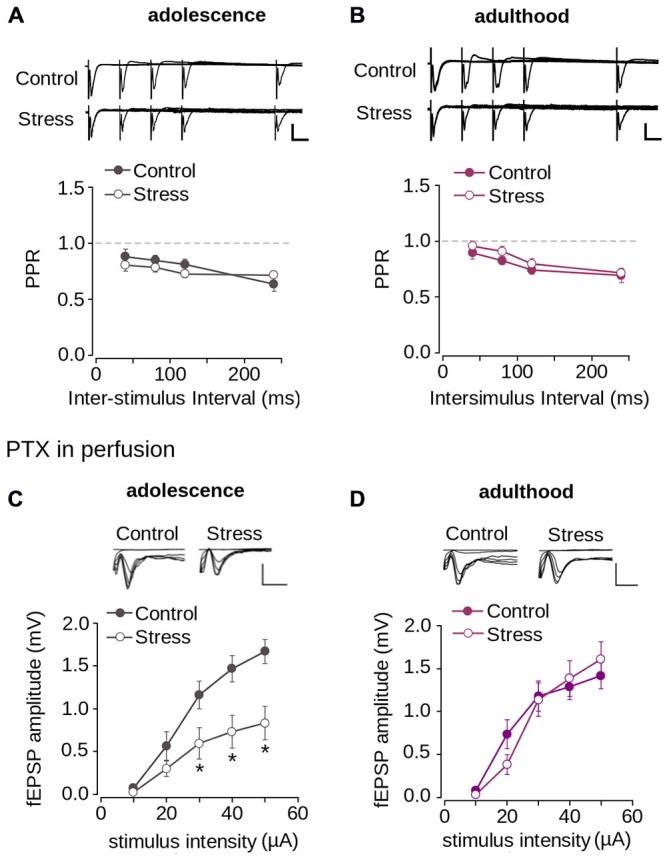
**Repeated stress did not affect paired-pulse depression and inhibitory synaptic transmission in layer 1 of mPFC. (A,B)** Upper panel, representative superimposed traces from control and stress groups evoked by PPS delivered at ISI of 20, 40, 80, and 120 ms during adolescence **(A)** and adulthood **(B)** (Scale bar: 1 mV; 10 ms). Lower panel, average PPR values between control and stress groups during adolescence **(A)** and adulthood **(B)**. Data are expressed as mean ± SEM. **(C,D)** Upper panel, superimposed representative traces of the fEPSP evoked under different stimulus intensities in the presence of PTX 1 μM for control and stress conditions during adolescence **(C)** and adulthood **(D)** (Scale bar: 1 mV; 5 ms). Lower panel, input-output relationship of fEPSP amplitude as function of stimulus intensity during PTX application for control and stress condition during adolescence **(C)** and adulthood **(D)**. Significant difference between control and stress: *p < 0.05; Bonferroni *post hoc* test. Data are expressed as mean ± SEM (4 animals per group; 17-18 slices per group).

Given that the reduction of synaptic transmission is attributed to decreased neurotransmitter release in the presynaptic domain (Zucker and Regehr, 2002), we evaluated the probability of neurotransmitter release using the PPS protocol. As shown in Figures 5A,B we found paired-pulse depression at all tested ISIs. There were no significant differences in the PPR values for control and stressed adolescent (Figure 5A; effect of repeated stress: *F*_(1,204)_ = 1.392; *p* = 0.239) and adult animals (Figure 5B; effect of repeated stress: *F*_(1,252)_ = 2.36; *p* = 0.125) at different ISIs. This suggests that the reduction in synaptic transmission in the mPFC induced by repeated stress was not related to alterations in the probability of neurotransmitter release.

The reduction of synaptic transmission in the mPFC could also be attributed to increased inhibitory transmission. To examine this possibility, we blocked inhibitory transmission by applying PTX, a GABA_A_R blocker. Figure 5C shows that the application of PTX did not abolish the reduction of the fEPSP amplitude of stressed animals compared to controls in adolescence (effect of repeated stress: *F*_(1,102)_ = 26.26; *p* < 0.0001). Bonferroni *post hoc* comparison showed significantly lower fEPSP amplitudes in the stressed group, measured in adolescence at 30, 40 and 50 μA, than in the control (Figure 5C). We analyzed the effect of repeated stress in adulthood and found no differences between control and stressed groups (Figure 5D; effect of repeated stress: *F*_(1, 156)_ = 0.065; *p* = 0.798). Altogether, the data suggest that the decrease in synaptic function in the mPFC induced by repeated stress was independent of inhibitory transmission, and that excitatory transmission was affected.

**Figure 6:**
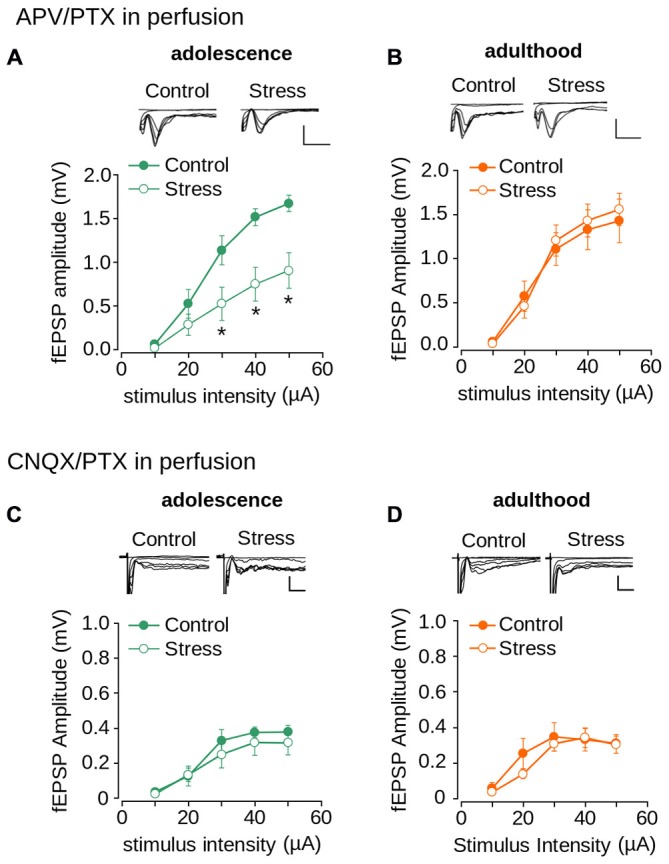
**Effect of repeated stress on AMPA/kainate receptor and NMDAR-mediated synaptic transmission in layer 1 of mPFC during adolescence and adulthood. (A,B)** Upper panel, representative superimposed traces of the fEPSP evoked under different stimulus intensities in the presence of PTX 1 μM and 50 μM APV for control and stress groups during adolescence **(A)** and adulthood **(B)** (Scale bar: 1 mV; 5 ms). Lower panel, input-output relationship of fEPSP amplitude as a function of stimulus intensity during co-application of PTX and APV for control and stress groups during adolescence **(A)** and adulthood **(B)**. Significant difference between groups (*p < 0.05; Bonferroni *post hoc* test). Data are expressed as mean ± SEM. **(C,D)** Upper panel, representative traces of the fEPSP evoked under different stimulus intensities in ACSF without Mg^2+^ in the presence of PTX 1 μM and 50 μM CNQX for control and stress groups during adolescence **(C)** and adulthood **(D)** (Scale bar: 0.5 ms; 10 ms). Lower panel, input-output relationship of fEPSP amplitude as a function of stimulus intensity during co-application of PTX and CNQX for control and stress groups. Data are expressed as mean ± SEM (n = 4 animals per group; 10-12 slices per group).

To evaluate the contribution of AMPA/kainate and NMDA receptors to repeated stress-induced impairment in synaptic transmission we pharmacologically isolated both NMDAR- and AMPA/kainate receptor-mediated transmissions. As shown in Figure 6A, the AMPA/kainate receptor-mediated fEPSP amplitude was significantly lower in the stressed animals than in the controls in adolescence (effect of repeated stress: *F*_(1,102)_ = 27.14; *p* < 0.0001). The Bonferroni *post hoc* comparison showed significantly lower AMPA/kainate mediated-receptor fEPSP amplitude in the stressed group at 30, 40 and 50 μA of stimulus intensity (Figure 6A). The reduced response in stressed animals returned to control levels in adulthood (Figure 6B; *F*_(1,108)_ = 0.092; *p* = 0.761). Subsequently, we evaluated the effect of repeated stress in NMDAR-mediated transmission. As shown in Figures 6C,D we found no differences in NMDAR-mediated fEPSP amplitudes between adolescent (Figure 6C; effect of repeated stress: (*F*_(1,96)_ = 1.859; *p* = 0.175) and adult (Figure 6D; effect of repeated stress: *F*_(1,90)_ = 1.18; *p* = 0.28) control and stressed group animals. These findings suggest that repeated restraint stress decreases AMPA/kainate receptor-mediated glutamatergic transmission in layer 1 of the mPFC, which returns to control levels in adulthood after stress-free period.

### Repeated Stress Reduced LTD During Adolescence and was Reversed at Adulthood

**Figure 7:**
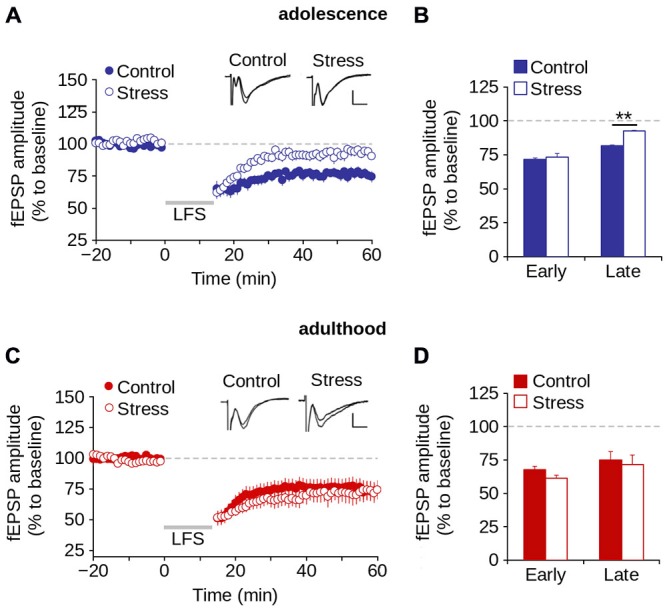
**Repeated stress-induced impairment of the LTD in layer 1 of mPFC during adolescence is reversed in adulthood. (A,B)** Time lapse of normalized fEPSP amplitude expressed as a percentage of the baseline for control and stress conditions during adolescence **(A)** and adulthood **(B)**. Inset: representative superimposed traces from control and stress groups before and after the application of the LFS protocol (indicated by the gray bar). Calibration bar: 0.5 mV; 5 ms. **(C,D)** Bar chart of the average normalized fEPSP amplitude recorded during the first 10 min (early-LTD) and last 10 min (late-LTD) after the application of the LFS protocol for control and stress conditions during adolescence **(C)** and adulthood **(D)** (**p < 0.05; Bonferroni *post hoc* comparison). Data are expressed as mean ± SEM (*n* = 4 animals per group; 10-11 slices per group).

We investigated the effect of repeated stress on LTD induction (early LTD) and expression (late LTD) in adolescence and adulthood (Malenka and Bear, 2004). Figure 7A shows that the application of LFS protocol induced LTD in both control and stress groups during adolescence. However, repeated stress reduced the magnitude of LTD (effect of repeated stress: *F*_(1,36)_ = 13.59; *p* < 0.001; Figures 7A,B). Specifically, we found significant differences between groups in the magnitude of late LTD (control = 76.3 ± 3.9%; stress = 92.1 ± 3.2%; *p* < 0.01; Bonferroni *post hoc* comparison) but not of early LTD (control = 66.4 ± 3.9%; stress = 73.6 ± 2.4%; *p* > 0.05; Bonferroni *post hoc* comparison).

We then evaluated the effect of repeated stress on LTD in adult rats. Figure 7C shows no significant effect of repeated stress (*F*_(1,25)_ = 0.474; *p* = 0.497) on the magnitude of LTD. We found no significant differences between groups in the magnitude of either early (control = 59.8 ± 5.5%; stress = 53.2 ± 3.7%; *p* > 0.05) or late LTD (control = 74.1 ± 6.5%; stress = 71.8 ± 9.1%; *p* > 0.05; Figure 7D). The data suggest that repeated stress impaired the expression, and not the induction of LTD in the mPFC during adolescence, and that LTD was recovered in stressed animals in adulthood after a stress-free period.

## Discussion

### Characterization of Synaptic Transmission and Plasticity in Layer 1 of the mPFC

We found that synaptic activity in layer 1 of the mPFC was mostly mediated by AMPA/kainate and NMDA receptors, which also displayed paired-pulse depression and NMDA-dependent LTD. However, under our working conditions, LTP was not induced. Similar results have been published previously related to the glutamatergic receptors involved in synaptic response (Hirsch and Crepel, 1990), and the NMDA-dependence of LTD (Otani et al., 1999). Similar results have also been reported with respect to the non-induction of LTP under comparable working conditions (Otani et al., 1998; Morris et al., 1999). The activation of dopaminergic receptors seems to be crucial for inducing LTP in mPFC slices (Kolomiets et al., 2009). In *in vivo* models, where dopaminergic fibers are preserved, LTP is commonly evoked in the mPFC, and blocking D1 receptor antagonists blocks LTP (Gurden et al., 2000; Coppa-Hopman et al., 2009). Thus, the lack of LTP observed in our experiments could be related to the loss of dopaminergic afferents to the mPFC.

The fEPSP recorded in layer 1 in our study may in part reflect the activation of deeper synapses in the same neuronal population due to collaterals from layer 1 contacting pyramidal cells in deeper regions. However, previous data have shown that horizontal fibers that cross layer 1 almost exclusively contact monosynaptically the apical tufts of pyramidal neurons of layers 2/3 and 5, with little activation of synapses in deeper layers (Cauller and Connors, 1994). The short-latency of the response, together with the absence of long-latency responses, strongly suggests that the fEPSP recorded by stimulation of layer 1 is related to activated synapses in the apical tufts of pyramidal neurons.

The apical dendritic tufts in the mPFC are innervated by horizontal fibers that arrive mainly from the mediodorsal thalamus, the amygdala and distal cortical areas (Bacon et al., 1996; Wang and Shyu, 2004). Importantly, apical dendritic tufts also receive recurrent inputs from local pyramidal neurons (Barbas and Rempel-Clower, 1997). Therefore, synapses in layer 1 of the mPFC receive not only long-range associational input, but also local cortical computations mediating complex cognitive processes (Roland, 2002). Given that chronic stress produces morphological dendritic atrophy in apical tufts of pyramidal neurons in the mPFC (Cook and Wellman, 2004; Radley et al., 2004, 2005, 2008; Liston et al., 2006; Brown et al., 2005; Izquierdo et al., 2006; Liu and Aghajanian, 2008), we suggest the function of superficial synapses is relevant to understand the effect of chronic stress on cognitive function mediated by the mPFC.

### Repeated Stress During Adolescence Decreases Synaptic Transmission and LTD in the mPFC

We found that 7 days of restraint stress during adolescence decreased fEPSP amplitude in layer 1 of the mPFC. This reduction is not attributed to differences in the amount of stimulated afferent fibers or changes in probability of neurotransmitter release, because we did not find differences in either the amplitude of the presynaptic volley or the magnitude of the PPR, respectively. Our results indicate that the repeated stress-induced decrease in basal synaptic transmission during adolescence is mediated by post-synaptic alterations in AMPA/kainate dependent transmission, but not because of alterations in GABA_A_ or NMDA receptors. It has been reported that chronic stress, as well as corticosterone administration, decreases both AMPA/kainate receptor-mediated transmission and the number of AMPA/kainate receptors in the mPFC by proteasome-mediated degradation (Yuen et al., 2012). Likewise, chronic treatment with corticosterone decreases the number of GluR2/3 subunits of AMPA/kainate receptors in the mPFC (Gourley et al., 2009). Thus, decreased AMPA/kainate receptor-mediated transmission could be related to reduced availability of AMPA/kainate receptors in the postsynaptic domain.

We also found that repeated stress during adolescence decreased the expression of LTD, but not its induction. Given that the induction of LTD in the mPFC seems to be dependent on NMDAR (Figure 2D), this result supports the view that repeated stress does not affect NMDAR-mediated transmission. Interestingly, AMPA/kainate receptors are crucial for the maintenance of NMDA-dependent LTD (Ehlers, 2000; He et al., 2011). The repeated stress-induced reduction of AMPA/kainate receptors-mediated transmission may decrease not only synaptic efficiency in the mPFC, but also the magnitude of late LTD in the mPFC in adolescence.

### The Reduction of Synaptic Transmission and LTD Induced by Repeated Stress in the mPFC During Adolescence were Reversed in Adulthood

We found that both basal synaptic transmission and LTD were recovered in adulthood after a stress-free period. Interestingly, the recovery of synaptic transmission and LTD was accompanied by a recovery of AMPA/kainate receptor-mediated transmission. Earlier studies showed that chronic stress-induced dendritic atrophy in the mPFC during adolescence was reversed in adulthood (Radley et al., 2005; Goldwater et al., 2009). In accordance with our results, Yuen et al. (2012) found that stress induced impairment of synaptic transmission in the mPFC was reversed 10 days after repeated stress, a change that was associated with alterations in glutamatergic function.

What processes are involved in the observed effects? The evidence suggests that stress response is more intense during adolescence (Pruessner et al., 2005; McCormick et al., 2010) because the glucocorticoids levels are elevated in the mPFC, which in turn induces synaptic alterations like dendritic atrophy and synaptic impairment (Cook and Wellman, 2004; Yuen et al., 2012). Several neuronal modifications related to maturation of the mPFC during adolescence have been described (Markham et al., 2007, 2013; Kolb et al., 2010). For example, synaptic pruning, a hallmark of the remodeling process associated with adolescence, is present in rats by PD31, with spine density in the mPFC decreasing thereafter until PND60 (Koss et al., 2014). NMDA receptors, as well as the AMPA receptor component GluA2, reach a maximum of expression around P28–30 and then decrease by P60 (Insel et al., 1990; Wang and Gao, 2009; Murphy et al., 2012). Dopaminergic responses of both excitatory and inhibitory neurons increase in the mPFC (O’Donnell, 2010). Indeed, during adolescence dopamine modulates the prefrontal glutamatergic and GABAergic response of pyramidal neurons and interneurons (Tseng and O’Donnell, 2004, 2007). These neuronal modifications sustain homeostatic synaptic plasticity and refine neural circuitry required to the complete development of the PFC (Selemon, 2013). These changes in the mPFC may permit the reversal or moderation of the effects of early experiences through late developmental processes. A high degree of plasticity may permit the recovery of neural function in response to environmental stimulus like chronic stress.

### Concluding Remarks

We previously found that repeated stress during adolescence impairs the recall of the extinction of conditioned fear, an impairment that was reversed in adulthood (Negrón-Oyarzo et al., 2014). The behavioral impairment and its subsequent reversal correlated with alterations in synaptic transmission in the mPFC (Negrón-Oyarzo et al., 2014). In the present work, we found that synaptic impairment in the mPFC induced by repeated stress in adolescence and the subsequent reversal in adulthood relate to a decrease and recuperation of AMPA/kainate receptor-mediated glutamatergic transmission and LTD, which supports the role of glutamatergic synaptic transmission and plasticity in behavioral functions in the mPFC.

Adolescence is often described as a developmental window of vulnerability to psychiatric disorders (Paus et al., 2008). However, it is unclear whether chronic stress-induced alterations in neural circuitry involved in affective and cognitive functions remain until adulthood after a stress-free period. Previous studies have shown that both neurophysiological and behavioral function supported by some neural systems, like the amygdala, do not recover in adulthood after a stress-free period (Vyas et al., 2004; Negrón-Oyarzo et al., 2014). In our present and previous studies (Negrón-Oyarzo et al., 2014) we have shown that alterations of behavioral tasks supported by the mPFC during adolescence associated with chronic stress were reversed in adulthood after a stress-free period. This suggests that the mPFC is able to remodel neural circuitry in response to repeated stress allowing for the recovery of behavioral functions.

## Author Contributions

IN-O designed research, performed the experiments and analyzed the results. AD-S and PMC supervised the performance of the experiments and data analysis. IN-O and PMC wrote the paper and all authors critically revised the manuscript.

## Conflict of Interest Statement

The authors declare that the research was conducted in the absence of any commercial or financial relationships that could be construed as a potential conflict of interest.
